# Comparison of cylindrical and tapered stem designs for femoral revision hip arthroplasty

**DOI:** 10.1186/s12891-020-03461-5

**Published:** 2020-06-29

**Authors:** Yu Zhang, Ye Zhang, Jian-Ning Sun, Zi-Jian Hua, Xiang-Yang Chen, Shuo Feng

**Affiliations:** 1 Department of Orthopedic Surgery, Affiliated Hospital of Xuzhou Medical University, 99 Huaihai Road, Xuzhou, 221002 Jiangsu China; 2 Department of Orthopedic Surgery, The Third Hospital of Hebei Medical University, 139 Ziqiang Road, Shijiazhuang, 050000 Hebei China

**Keywords:** Femoral revisions, Hip arthroplasty, Cylindrical stem, Tapered stem, Complications

## Abstract

**Background:**

Both cylindrical and tapered stems are commonly used in revision total hip arthroplasty. However, whether the geometry of prosthesis stem has an effect on patient prognosis is unclear. We assume that the tapered stem results in better clinical outcome than the cylindrical stem.

**Methods:**

A multicenter review of 120 femoral revisions with Paprosky I, II, and III defects using cobalt chrome cylindrical stem (54 hips) or titanium tapered stem (66 hips) was performed with an average follow-up of 6 years. Demographic data were comparable between groups.

**Results:**

No significant group differences were found in surgery time, bleeding volume, postoperative Harris Hip Score, level of overall satisfaction, and 8-year cumulative survival. However, intraoperative fractures occurred significantly less in the tapered group (4.5%) than in the cylindrical group (14.8%), and stem subsidence was significantly less in the tapered group (2.17 mm) than in the cylindrical group (4.17 mm). A higher ratio of bone repair and lower bone loss were observed in the tapered group compared with the cylindrical group. The postoperative thigh pain rate was higher in the cylindrical group (12.9%) than in the tapered group (4.5%).

**Conclusion:**

Both cylindrical stem and tapered stem can achieve satisfactory mid-term clinical results in revision total hip arthroplasty. The tapered stem has better bone restoration of proximal femur, lower incidence of intraoperative fractures, and lower postoperative thigh pain rate compared with the cylindrical stem.

## Background

As one of the most successful surgical operations in the twentieth century, total hip arthroplasty (THA) can significantly reduce pain, improve function, and correct hip deformities, thereby improving patients’ quality of life. With the increase of primary THA worldwide, especially in younger patient populations, the number of cases requiring revision hip arthroplasty has been increasing due to aseptic loosening, fracture, and dislocation of prosthesis. The Swedish Hip Arthroplasty Register recently reported that the percentage of revisions has more than tripled since the 1990s [1]. Femoral stem revision can be challenging due to the loss of bone mass in the proximal femur and the quantity and quality of the remaining host bone. Richards et al. [2] believe that the four main objectives of femoral revision are to achieve long-term implantation and fixation, improve patients’ quality of life, reduce complications, and maintain or restore the bone mass of the proximal femur.

Cemented prostheses are mostly used in the early femoral revision of the hip joint. However, due to severe bone defect and sclerosis of the medullary cavity, the anchorage between the cement and the bone cortex decreases, which leads to higher early loosening rate; thus, the use of these prostheses is gradually being abandoned [3–5]. At present, more attention has been paid to the application of biological long-stem prosthesis in revision surgery. In North America, extensively coated cylindrical stem is widely used. The distal part of the prosthesis is cylindrical, and the prosthesis can cross the defective area of the proximal femur. With the help of the close compression between the prosthesis and the distal femoral medullary cavity, the initial rotation stability and axial stability of the prosthesis can be achieved, thereby creating conditions for secondary bone growth. Several studies have shown that good clinical and imaging results can be obtained when extensively coated cylindrical stem is used in revision THA [2, 6, 7]. However, some scholars reported that the use of such prostheses is associated with severe postoperative thigh pain (8–9%) and severe stress shielding of the proximal femur (6–7.6%) [2]. In some patients with Paprosky type III femoral defects, the failure rate of these femoral stems is high [8].

Another widely used cementless prosthesis for femoral revision was first reported by Wagner. The distal stem of this prosthesis has a tapered geometric design. It has eight sharp lateral ridges on the surface and grooves formed by adjacent lateral ridges. It has been widely used in Europe and has been reported to reduce the incidence of bone resorption caused by stress shielding in the proximal femur and achieve good spontaneous bone regeneration in the proximal femur. Gutierrez et al. [9] suggested that spontaneous bone repair in the proximal femur may be related to the tapered design, titanium alloy material, and good biocompatibility of the rough surface. However, some scholars reported that the tapered stem prosthesis has a high rate of subsidence and dislocation. Femoral prosthesis subsidence occurs in up to 35% of patients [10].

Therefore, in the revision of hip femoral prosthesis, the effect of prosthesis geometry on the long-term clinical outcome of patients is still unclear. Although many reports on the clinical efficacy of single stem are available, few studies have investigated the mid- and long-term clinical results of stems with two different geometric shapes. Thus, the present retrospective study compared the mid- and long-term clinical outcomes, imaging results, postoperative complications, and survivorship of cylindrical stem and tapered stem prostheses for femoral revision to determine whether the tapered distal geometry of the femoral prosthesis is superior to that of the cylindrical stem.

## Methods

### Patient selection

Patients who underwent revision THA with extensively coated cylindrical stem (Solution Stem, DePuy, USA) and extensively coated tapered stem (Wagner SL, Zimmer, USA) in two institutions from January 2009 to June 2018 were reviewed. These stems are both monoblock. The extensively coated cylindrical stem has a distal cylindrical geometry, whereas the grit-blasted tapered stem has a distal tapered geometry. The cylindrical stem has a cobalt chrome shaft with a circular cross section and a beaded porous coating to allow for bone ingrowth. The tapered stem has a titanium shaft with a circular cross section and a 2° taper. It has flutes for rotational stability and a grit-blasted surface texture for bone on-growth. This retrospective study was approved by the ethical committee of our institution, and all methods were performed in accordance with the relevant guidelines and regulations. Informed consent was obtained from all patients.

A total of 127 patients were initially identified. Seven patients (eight hips) were lost to follow-up, and five patients (five hips) died of causes unrelated to their operation. The remaining 115 patients (120 hips) were analyzed. According to the type of femoral prosthesis, the patients were divided into the cylindrical group (54 hips) and tapered group (66 hips). The general data of the two groups are shown in Table 1. No significant difference was observed in the preoperative data between the two groups, and the two groups were comparable.

**Table 1:** Comparison of basic data between the two groups

classification	cylindrical group	tapered group	*P* value
Age (years)	68.3 ± 7.0(49 ~ 81)	67.7 ± 7.9(50 ~ 83)	0.481
Gender (female/male)	28/26	30/36	0.860
BMI(kg/m^2^)	26.1 ± 3.0 (19.00 ~ 32.00)	26.0 ± 2.5 (20.74 ~ 31.99)	0.860
Initial replacement to repair time (months)	11.5 ± 4.8 (1 ~ 21)	10.9 ± 6.6 (0.08 ~ 25)	0.370
reasons for revision(n)			0.583
Aseptic loosening	49	57	
Periprosthetic fractures	3	3	
Dislocation	2	6	
Paprosky femoral defect (n)			0.347
I	10	12	
II	27	36	
IIIA	16	15	
IIIB	1	3	
ASA classification(n)			0.168
I	5	6	
II	41	48	
III	8	12	
Combined acetabular revision(n)	47	63	0.071
VAS score (score)	7.6 ± 1.3 (6 ~ 10)	7.5 ± 1.1 (6 ~ 10)	0.982
Harris score (score)	41.1 ± 6.1 (29 ~ 52)	40.1 ± 6.6 (27 ~ 52)	0.423

### Surgical methods

The two groups were treated with the same perioperative measures, including the use of antibiotics and the prevention of venous thrombosis. Preparations for antibiotic skin test and intravenous drip were performed half an hour before the operation. All patients received general anesthesia and were incised through the original extended posterolateral approach. If the femoral prosthesis was difficult to remove, extended trochanteric osteotomy was performed. After operation, the affected limb was maintained in a neutral and abducted position, and the drainage tube was removed 24–48 h after operation. Routine blood tests, erythrocyte sedimentation rate, C-reactive protein, and postoperative double hip joint radiographs were reviewed. After the patients awoke from anesthesia, they were instructed to conduct quadriceps femoris isometric contraction and ankle flexion and extension. Under the guidance of a physiotherapist on the second day after operation, the patients performed partial weight-bearing, and whole body weight-bearing was achieved within 6 weeks after operation. The patients who suffered from fracture during operation were treated with steel wire bandage or internal fixation plate. They were confined to bed for 6 weeks and prevented from performing weight-bearing activities. After re-examination, the patients were still prevented from performing weight-bearing activities. All patients were followed up regularly before operation; 6 weeks, 3 months, and 6 months after operation; and every year thereafter to evaluate for pain, hip function, and imaging results.

### Clinical assessment

Pain, range of motion, walking, stair climbing, limping, and daily activities were assessed by using the Harris Hip Score before and during each follow-up. The Visual Analogue Scale (VAS) was used to evaluate the degree of thigh pain before and after operation. Patients who could not go to the hospital after surgery were followed up through telephone calls and emails. At each follow-up, patients’ satisfaction with their surgical results was subjectively divided into five levels [11]: very unsatisfactory, unsatisfactory but tolerable, neutral, satisfied, and very satisfied. Data from the last follow-up were used in the analysis. The operation time, bleeding volume (intraoperative bleeding + postoperative drainage), blood transfusion volume, hospitalization time, and complications (intraoperative fracture, periprosthetic fracture, dislocation, infection) were recorded.

### Radiographic assessment

The bone defect of the femur was evaluated by using the Paprosky classification [12]. In comparing the initial X-ray images with the final X-ray images to evaluate the prosthesis subsidence, the measurement method of Callaghan et al. [13] was used to evaluate the prosthesis subsidence by measuring the vertical movement distance between the top of the femoral prosthesis and the greater trochanter. The stability of the femoral prosthesis was evaluated by using the standard assessment method proposed by Engh et al. [6]. It could be divided into bone ingrowth fixation, stable fibrous fixation, and unstable prosthesis. Bone ingrowth fixation was defined as an implant with no subsidence and minimal or no radio-opaque line formation around the stem. Stable fibrous fixation was defined as an implant with no progressive migration and < 1 mm extensive radio-opaque line formation around the stem and parallel to the stem. An unstable prosthesis was defined as one with definite evidence of either progressive subsidence or migration within the canal and is at least partially surrounded by divergent radio-opaque lines. To evaluate the difference in the proximal femoral bone stock between the initial postoperative and most recent follow-up radiographs, changes in proximal femoral bone reserve were classified according to the criteria described by Bohm and Bischel [14]: A (increasing defects), B (constant defects), or C (osseous restoration). If a difference was noted, the dates of the radiographs were revealed to ascertain if this represented type A or C. Stress shielding was assessed by using the methods of Engh and Bobyn [15]: In degree I, the femoral calcar becomes round and blunt, the bone density decreases, and the femoral calcar is atrophied. In degree II, the reduction of bone mineral density involves the trochanter on the basis of degree I. In degree III, the bone mineral density of the proximal isthmus is decreased. In degree IV, the cortical bone density extending to the isthmus is decreased.

The radiographic assessments were interpreted by one fellowship-trained academic musculoskeletal radiologist who has 20 years of experience in interpreting hip X-ray images. Engh et al. [16] reported on the reliability of radiographic evaluation for femoral bone loss and noted J values of 0.58 and 0.74 for interobserver reliability and intraobserver reliability, respectively.

### Statistical analysis

The data and charts were analyzed and processed by IBMS SPSS Statistics 19.0 software. Continuous variables were analyzed using independent samples t-test. Categorical variables were analyzed using the Pearson chi-square or Fisher exact tests. Kaplan–Meier survivorship analyses were used with the endpoint defined as any reoperation for any reason. At both sides, α was set at 0.05, and *P* < 0.05 was considered statistically significant.

## Results

### Basic conditions of surgery

No significant difference was observed in the operation time, length of stay, blood loss, and blood transfusion between the two groups (*P* > 0.05). A comparison of the intraoperative data between the two groups is shown in Table 2.

**Table 2:** Comparison of intraoperative data between the two groups

classification	cylindrical group	tapered group	*P* value
operative time (minutes)	234.6 ± 48.3 (120 ~ 330)	229.3 ± 62.6 (120 ~ 385)	0.399
length of stay (days)	20.9 ± 4.6 (12 ~ 34)	20.5 ± 4.9 (10 ~ 40)	0.451
Intraoperative blood loss (ml)	1240.7 ± 306.2 (500 ~ 2000)	1210.6 ± 491.4 (300 ~ 2200)	0.441
Postoperative drainage (ml)	536.2 ± 88.0 (310 ~ 754)	520.8 ± 115.6 (315 ~ 774)	0.203
Total blood loss (ml)	1784.4 ± 317.7 (984 ~ 2494)	1748.1 ± 514.5 (882 ~ 3525)	0.518
Blood transfusion volume (ml)	711.1 ± 276.5 (400 ~ 1600)	709.1 ± 320.0 (400 ~ 1600)	0.773
Wire binding (n)	24	24	
Allograft bone plate(n)	5	6	
Intraoperative fractures(n)	8	3	
Extended trochanteric osteotomy (n)	10	9	

### Clinical results

**Fig. 1:**
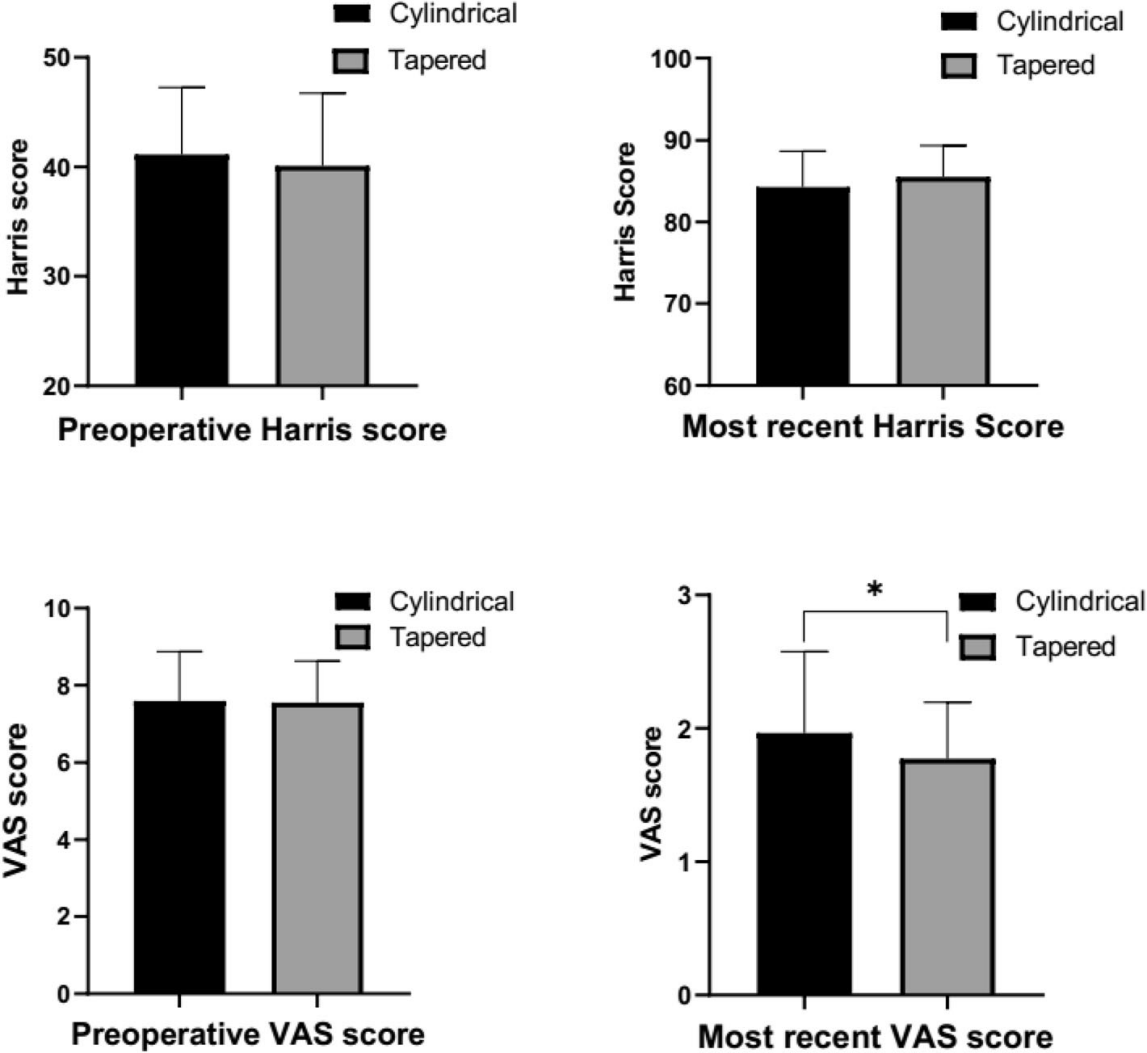
Comparison of the preoperative and postoperative Harris score and VAS score between the two groups

A total of 120 patients were followed up with an average of 74.8 months (12–114 months). At the last follow-up, the Harris Hip Score in the cylindrical and tapered groups increased from 41.1 ± 6.1 to 84.3 ± 4.4 (*P* < 0.05) and from 40.1 ± 6.6 to 85.5 ± 3.8 (P < 0.05), respectively. The VAS score in the cylindrical and tapered groups decreased from 7.6 ± 1.6 to 2.0 ± 0.4 (P < 0.05) and from 7.5 ± 1.2 to 1.8 ± 0.2 (P < 0.05), respectively. At the last follow-up, no significant difference was observed in the Harris Hip Score between the two groups (*P* > 0.05), but the VAS score of the cylindrical group was higher than that of the tapered group (*P* = 0.047) (Fig. 1). Moreover, no significant difference was observed in the overall satisfaction of recent follow-up results between the cylindrical group (87.3%) and the tapered group (90.1%) (Table 3).

**Table 3:** Level of satisfaction at the most recent follow-up

level of overall satisfaction	Cylindrical group (*N* = 54)	tapered group (*N* = 66)
Very satisfied	29	43
Satisfied	18	17
Neutral	3	2
Dissatisfied	2	2
Very dissatisfied	2	2

### Radiographic results

**Fig. 2:**
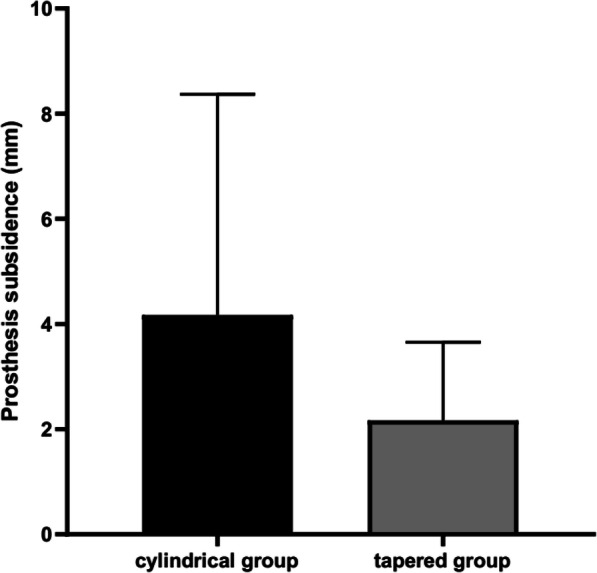
Comparison of the prosthesis subsidence between the two groups at the last follow-up (P < 0.05)

At the last follow-up, prosthesis subsidence in the cylindrical and tapered groups was 0–15 mm with an average of (4.17 ± 4.20) mm and 0–8 mm with an average of (2.17 ± 1.49) mm, respectively (Fig. 2). A significant difference was observed in prosthesis subsidence between the two groups. All prosthesis subsidence stopped within 1 year after operation. Ten hips in the tapered group (15.2%) and seven hips in the cylindrical group (13.0%) experienced subsidence of more than 5 mm (*P* > 0.05). Among the 10 hips in the tapered group, eight had type IIIA femoral defects, and two had type IIIB femoral defects. Among the seven hips in the cylindrical group, six hips had type IIIA femoral defects, and one had a type IIIB femoral defect.

In the cylindrical group, 45 hips (84.1%) were fixed by bone growth, seven (13.0%) were fixed by fibers, and one (1.8%) was unstable. In the tapered group, 64 hips (98.4%) were fixed by bone growth, one (1.5%) was fixed by fibers, and one (1.5%) was unstable. The failure rate of osseointegration (fibrous or unstable) in the cylindrical group was significantly higher compared with that in the tapered group (*P* < 0.05).

In the imaging changes of proximal femoral host bone, the proportion of bone repair type in the tapered group (39.4%) was significantly higher than that in the cylindrical group (7.4%, P < 0.05), and the proportion of bone loss type in the tapered group (13.6%) was significantly lower than that in the cylindrical group (43.6%, *P* < 0.05; Table 4).

**Table 4:** Radiographically evident changes to the proximal femur host bone stock

Changes to the proximal femur host bone stock	cylindrical group (*N* = 54)	tapered group (*N* = 66)	P value
Type A(bone loss)	23	9	0.000
Type B (no change)	27	31	0.741
Type C(bone restoration)	4	26	0.000

In the cylindrical group, 21 hips (38.9%) had stress-shielded bone resorption of degrees I and II in the greater trochanter, which was manifested by femoral moment atrophy or decreased cortical bone mineral density from the sharply blunt margin to the trochanter level. However, in the tapered group, nine hips (13.6%) had stress-shielded bone resorption of degrees I and II in the greater trochanter. A significant difference was observed between the two groups (*P* < 0.05; Table 5).

**Table 5:** Comparison of postoperative data between the two groups

classification	cylindrical group (*N* = 54)	tapered group (*N* = 66)	*P* value
Stress-shielded bone resorption	21	9	0.001
Postoperative thigh pain	7	3	0.097
Subsidence of more than 5 mm	7	10	0.732
Paprosky I	0	0	
Paprosky II	0	0	
Paprosky IIIA	6	8	
Paprosky IIIB	1	2	
Stem length			
190(mm)	39	16	
225(mm)	–	31	
260(mm)	15	–	
265(mm)	–	19	
305(mm)	–	0	
190–230(mm)	39	47	0.903
230–270(mm)	15	19	0.903
>270(mm)	0	0	

### Survivorship

**Fig. 3:**
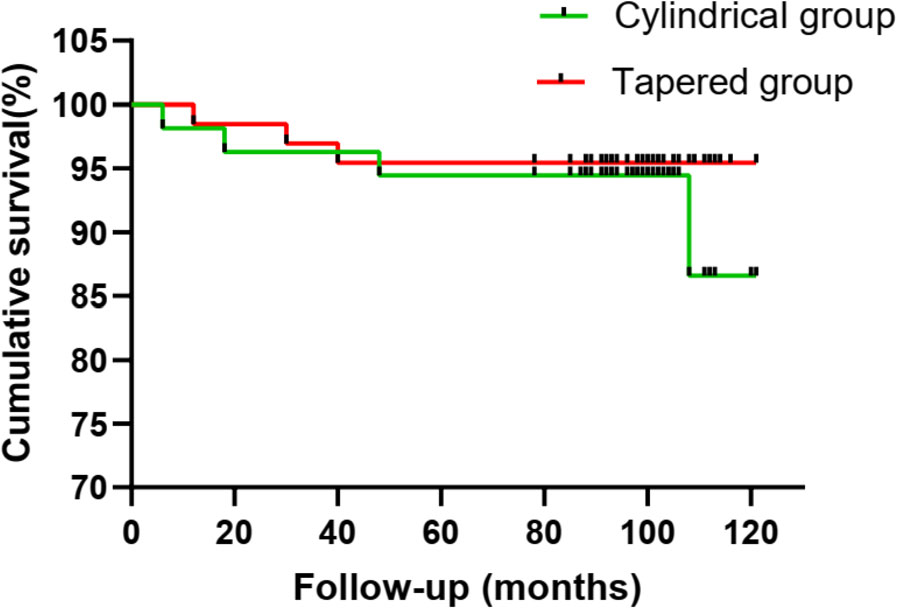
Kaplan-Meier survival analysis with the endpoint defined as any reoperation because of septic or aseptic complications

The 8-year cumulative survival rate was defined as the end point of any reoperation for any reason. The 8-year cumulative survivorship of the cylindrical and tapered stems was 94.43% (95% confidence interval [CI], 86.13–97.82%) and 96.69% (95% CI, 91.39–98.75%), respectively. No significant difference was observed between the two groups (Fig. 3).

### Postoperative complications

**Fig. 4:**
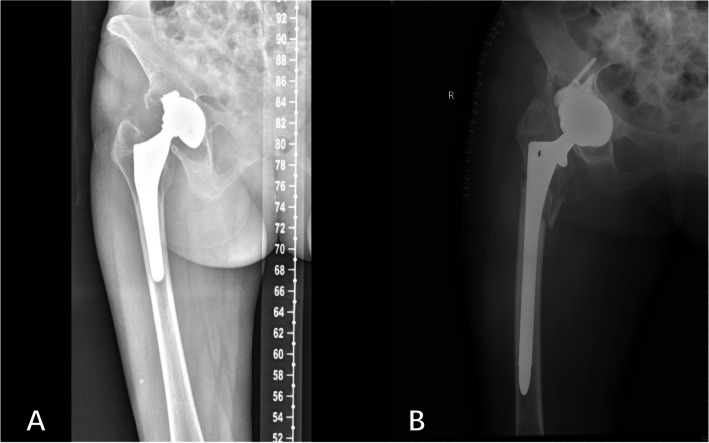
Preoperative and postoperative radiographs of cylindrical stem with intraoperative fractures

**Fig. 5:**
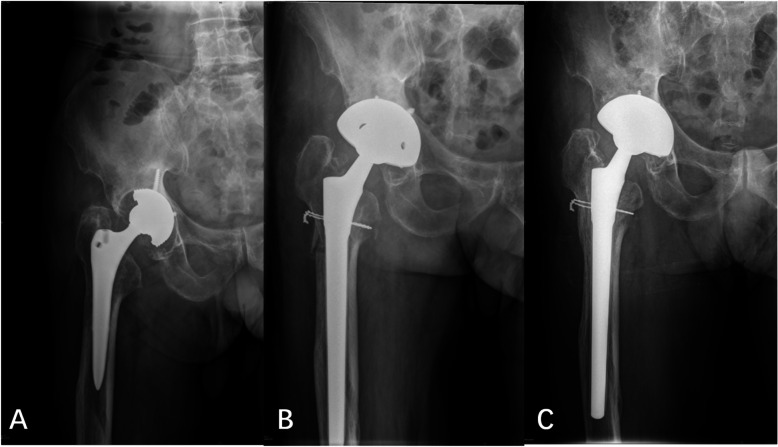
Preoperative and postoperative radiographs of tapered stem with intraoperative fractures

Intraoperative fractures were found in eight cases (14.8%, Fig. 4) in the cylindrical group and three cases (4.5%, Fig. 5) in the tapered group. A significant difference was observed between the two groups (P < 0.05). In the cylindrical group, five cases had femoral trochanteric fractures (with steel wire binding), and three cases had femoral shaft fractures (with steel wire binding). All fractures were healed. In the tapered group, three cases had femoral shaft fractures (with steel wire binding), and the fractures healed after operation.

Periprosthetic fractures were observed in two cases (3.7%) in the cylindrical group and one case (1.5%) in the tapered group (*P* > 0.05). These fractures were treated with open reduction and internal fixation.

Two cases (3.7%) of hip dislocation were observed in the cylindrical group after operation. One patient underwent cup exchange with recurrent instability, whereas the other was treated with closed reduction, regaining hip stability. Two cases (3.0%) of hip dislocation occurred in the tapered group (P > 0.05). One case of frequent dislocation after closed reduction was treated with replacement of lining and femoral head size.

**Fig. 6:**
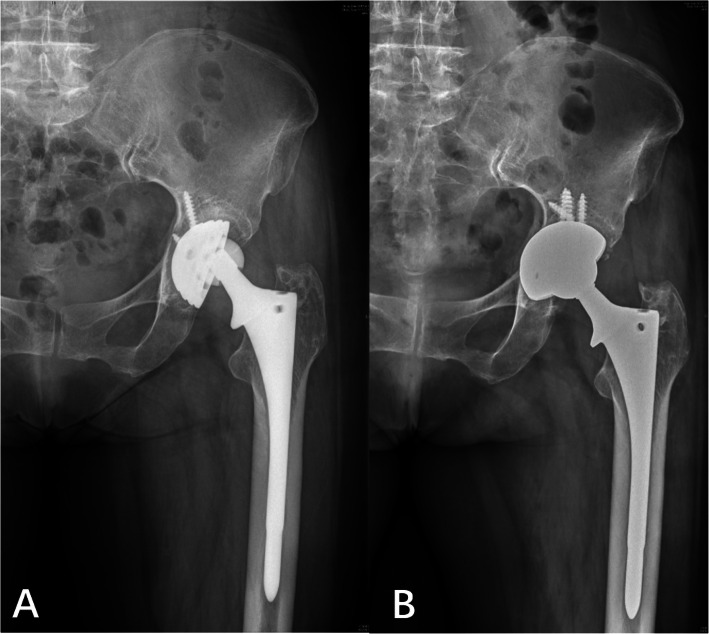
Postoperative radiographs of cylindrical stem with osteolysis around cup and re-revision with cup exchange

One hip in the cylindrical group was revised due to osteolysis around the cup 9 years after surgery (Fig. 6). The last follow-up radiography showed osteolysis around the cup, and cup revision arthroplasty was conducted for an osteolytic lesion.

Three out of 66 cases in the tapered group had mild thigh pain after operation (VAS score l–3), but most of the symptoms disappeared 1 year after operation. Seven out of 54 cases in the cylindrical group had mild thigh pain (VAS score 1–4), 2 of which developed persistent thigh pain. The rate of postoperative thigh pain was higher in the cylindrical group (12.9%) than in the tapered group (4.5%) (*P* < 0.05).

## Discussion

THA can successfully relieve pain and restore function in patients with advanced hip disease. However, complications including prosthetic loosening, infection, and fracture may require revision surgery. Preoperative planning and appropriate implant selection are critical for successful and lasting outcomes of THA revision. Damaged femurs usually have very little support at the proximal metaphysis but only limited support in the diaphysis. Severe femoral defects and changes in the shape of the femur pose challenges to adequate fixation in revision THA.

During the revision of non-cemented femoral prosthesis, different degrees of prosthesis subsidence will occur after the operation, and most of them transpire in the first year after the operation. The main reason is the insufficient press-fit between the prosthesis and the femoral medullary cavity during the operation, and subsidence can occur when the weight is loaded after the operation. In this study, femoral prosthesis with cylindrical stem and femoral prosthesis with tapered stem also had different degrees of subsidence after operation. At the last follow-up, the average subsidence of the tapered group was 2.17 mm (0–8 mm), which was significantly improved compared with that in the cylindrical group 4.17 mm (0–15 mm). All prosthesis subsidence stopped within 1 year after operation, which may be in the process of stem sinking. With the increase in the diameter of the proximal end of the tapered stem, the fixed strength of the tapered stem will be greater, and the tapered stem needs higher load than the cylindrical stem to produce settlement. Russell [17] reported that tapered stems required higher loads to produce subsidence than cylindrical stems in a revision THA model. Average loads to produce 150 μm of displacement with a 3-cm segment bone model were higher for the tapered stem than for the cylindrical stem (393 N vs. 221 N). Average loads to produce failure (> 4-mm subsidence) were also higher for tapered stems with a 3-cm segment (1574 N vs. 500 N). Revision tapered stems require a minimum intact segment of 1.5–2.5 cm to obtain adequate initial fixation stability. In case of severe bone loss, the reconstructed tapered stem has better initial fixation stability than the cylindrical stem. In the biomechanical comparison of tapered and cylindrical distal geometric structures in cadaveric models, the axial and rotational displacements of tapered designs are smaller when they are subjected to synchronous axial and torsional loads.

The stress shielding effect around the prosthesis has attracted increasing attention in the widely coated cylindrical stem. In this study, stress-shielded bone resorption of degrees I and II was observed in 21 hips of the cylindrical group. Kang [7] reported the revision of 45 hips with widely coated cylindrical stem. The average follow-up period was 12 years. The incidence of bone resorption in the proximal femur was 59.6% in 3 years, 65.4% in 5 years, and 67.3% in 10 years. Engh [18] found that proximal femoral bone resorption occurred more frequently in women, patients with low cortical index, and large-diameter femoral stem. Weede n[19] suggested that severe proximal femoral bone resorption is associated with preoperative osteoporosis and the use of large-diameter femoral stem. The shape of the prosthesis is an important factor to determine the degree of stress shielding. A tapered design femoral stem can be wedged into the femur to achieve stability. This kind of prosthesis can effectively reduce the stiffness of the prosthesis compared with the cylindrical stem fixed by backbone rubbing. The degree of stress shielding is lighter, and the stress distribution of the proximal femur is higher. Different materials are also a factor for the stress shielding. Titanium has a lower modulus of elasticity compared with cobalt-chromium, resulting in reduced femoral component stiffness for an equivalent diameter stem [2]. By reducing the modulus mismatch between the femoral component and the host bone, titanium stems may result in less proximal femoral stress shielding, particularly for small-diameter stems. Because stiffness is a function of the radius raised to the power of 4, this effect is less common in large-diameter stems.

Spontaneous reconstruction of the proximal femur accompanied by early bone mass recovery in revision THA with tapered stem has been reported in a mid- and long-term follow-up study [20]. In the imaging changes of proximal femoral host bone of this study, the proportion of bone repair type in the tapered group (39.4%) was significantly higher than that in the cylindrical group (7.4%), and the proportion of bone loss type in the tapered group (13.6%) was significantly lower than that in the cylindrical group (43.6%). Sandiford [21] followed up 104 patients who underwent Wagner SL femoral stem revision for 2 years. The bone remodeling rate was 47%. Bone remodeling could be observed as early as 3 months after operation, and no bone resorption occurred in the proximal femur. Regis et al. [20] followed up 41 patients with Wagner SL femoral stem revision for an average of 13.9 years. Approximately 63.9% of the patients showed proximal femoral bone remodeling, and 94.4% of the cortical bone thickness did not decrease at the last follow-up compared with that immediately after surgery. Gutierrez [9] suggested that the spontaneous bone repair of the proximal femur may be related to factors such as the tapered shape of Wagner SL prosthesis and titanium alloy material. According to Wolff’s law, bone growth is affected by mechanical stimulation, and the bone structure is changed. Stress shielding can lead to bone resorption and remodeling. A previous study [22] has shown that under the same degree of bone defect, the stress distribution in the bone defect area of the tapered group is higher than that of the cylindrical group, which may be conducive to bone reconstruction at the proximal femoral bone defect area. The stress of the widely coated cylindrical stem in the bone defect area is lower, which may lead to bone resorption in the bone defect area and aggravate bone loss in the proximal femur.

Another controversy over the cylindrical stem is thigh pain. In this study, seven of 54 cases (12.9%) in the cylindrical group developed mild thigh pain (VAS score 1–4) while walking after surgery, and two of them developed persistent thigh pain. In the tapered group, three out of 66 cases (4.5%) had mild thigh pain (VAS score l–3) while walking after surgery, but most of the symptoms disappeared after 1 year. Kang [7] followed up 45 hip revision patients for an average of 12 years. The incidence of thigh pain was initially 15.6%. The pain disappeared 3 years after operation. Paprosky [23] reported that patients with osteoporosis and femoral bone deficiency were more likely to have thigh pain. Some scholars [7] believe that insufficient prosthesis stability and stiffness mismatch between the bone and the prostheses are mainly responsible for the thigh pain. The former usually causes pain immediately after weight-bearing exercise. However, most of the pain will improve within 2 years, which is possibly due to internal fixation of stable fibers in the stem. The latter often manifests pain at the end of the prosthesis and is post-motion rather than initial. In material mechanics, the product of the elastic modulus of the material and geometric properties of the corresponding cross section is expressed as stiffness. In this study, the fibrous fixation in the cylindrical group (13.0%) was significantly higher than that in the tapered group (1.5%). Moreover, the cylindrical stem is cobalt-chromium alloy, whereas the tapered stem is titanium alloy. The stiffness of cobalt-chromium alloy prosthesis (diameter < 15 mm) is 3–5 times higher than that of the femoral shaft. Its elastic modulus is twice higher than that of titanium alloy. Cobalt-chromium alloy prosthesis shows greater stiffness than titanium alloy prosthesis [24, 25]. Moreover, the stress distribution is related to material properties and geometric shape of the object. This explains why persistent postoperative thigh pain frequently occurs in the cylindrical stem than in the tapered stem.

We acknowledge some limitations of this study. First, this retrospective study has a non-randomized design, the patients were treated by different surgeons in different institutions, and the number of patients in the two groups was low. Generally, a minimum of 2 years of follow-up on all patients is preferred. However, we think that 12 months is sufficient to determine subsidence and outcomes. Second, the implants used in this study were obtained from two different manufacturers. The cylindrical stem is cobalt-chromium alloy, whereas the tapered stem is titanium alloy. This study failed to control material confounding factor. The influence of material factors on the results cannot be excluded. Future studies should consider a multivariate analysis to control for bone loss classification, BMI, and age when reporting outcome metrics. Finally, the subsidence measured by imaging markers may not be as accurate as other techniques.

We conclude that cylindrical and tapered stems can achieve satisfactory mid-term clinical results in revision THA with the latter exhibiting better bone restoration of the proximal femur, lower incidence of intraoperative fractures, and lower rate of postoperative thigh pain than the former.

## Data Availability

We do not wish to share our data due to individual privacy, and according to the policy of our hospital, the data should not be shared to others without permission.

## Abbreviations

THA: Total hip arthroplasty
ETO: extended trochanteric osteotomy;
VAS: Visual Analogue Scale
